# A nested association mapping population identifies multiple small effect QTL conferring resistance against net blotch (*Pyrenophora teres* f. *teres*) in wild barley

**DOI:** 10.1371/journal.pone.0186803

**Published:** 2017-10-26

**Authors:** Thomas Vatter, Andreas Maurer, Doris Kopahnke, Dragan Perovic, Frank Ordon, Klaus Pillen

**Affiliations:** 1 Institute for Resistance Research and Stress Tolerance, Julius Kuehn-Institute, Federal Research Centre for Cultivated Plants, Quedlinburg, Germany; 2 Institute of Agricultural and Nutritional Sciences, Chair of Plant Breeding, Martin Luther University Halle-Wittenberg, Halle, Germany; Western Australia Department of Agriculture and Food, AUSTRALIA

## Abstract

The net form of net blotch caused by the necrotrophic fungus *Pyrenophora teres* f. *teres* is a major disease of barley, causing high yield losses and reduced malting and feed quality. Exploiting the allelic richness of wild barley proved to be a valuable tool to broaden the genetic base of resistance of modern elite cultivars. In this study, a SNP-based nested association mapping (NAM) study was conducted to map QTL for *P*. *teres* resistance in the barley population HEB-25 comprising 1,420 lines derived from BC_1_S_3_ generation. By scoring the percentage of infected leaf area followed by calculation of the average ordinate (AO) and scoring of the reaction type (RT) in two-year field trials a large variability of net blotch resistance across and within families of HEB-25 was observed. Genotype response to net blotch infection showed a range of 48.2% for AO (0.9–49.1%) and 6.4 for RT (2.2–8.6). NAM based on 5,715 informative SNPs resulted in the identification of 24 QTL for resistance against net blotch. Out of these, six QTL are considered novel showing no correspondence to previously reported QTL for net blotch resistance. Overall, variation of net blotch resistance in HEB-25 turned out to be controlled by small effect QTL. Results indicate the presence of alleles in HEB-25 differing in their effect on net blotch resistance. Results provide valuable information regarding the genetic architecture of the complex barley-*P*. *teres* f. *teres* interaction as well as for the improvement of net blotch resistance of elite barley cultivars.

## Introduction

The net form of net blotch caused by the necrotrophic fungus *Pyrenophora teres* f. *teres* is a major disease of barley worldwide. Infections can cause high yield losses typically ranging from 10 to 40% with the potential to result in total yield loss if susceptible cultivars are grown [1, 2]. Furthermore, infection results in a reduction of kernel size, plumpness, and bulk density, negatively affecting malting and feed quality [3].

Typical disease symptoms are transverse and longitudinal streaks, forming a net-like pattern of necrosis on barley leaves often accompanied by chlorosis [1]. Severe infection ultimately results in death of leaves in case of susceptible cultivars. *P*. *teres* f. *teres* can survive on kernels and barley debris in the field [4]. As a consequence, reduced or zero tillage has significantly increased the incidence of *P*. *teres* f. *teres* [5]. Although *P*. *teres* f. *teres* can be controlled by agricultural practice, e.g. wide crop rotation and ploughing or via fungicide application [4, 6], focus should be placed on breeding for durable resistance as a cost effective, environmental, and consumer-friendly approach.

No cultivars with complete resistance to *P*. *teres* f. *teres* have been identified up to now in Germany [7] but cultivars showing a low infection or slow disease development compared to susceptible cultivars are known [8]. The highly variable nature of *P*. *teres* f. *teres* and the influence of the developmental stage [9–15] turn the development of cultivars with improved resistance to *P*. *teres* f. *teres* into a challenging task. Numerous studies focusing on resistance of barley to *P*. *teres* f. *teres* resulted in the identification of a high number of QTL located on all barley chromosomes (reviewed in [4, 14–21]). These studies revealed net blotch resistance to be inherited mostly in a quantitative manner, especially in the adult plant stage. However, several dominant and recessive major genes were identified as well [6, 11, 19, 22–29]. Especially chromosome 6H turned out to harbor a high number of QTL and most of the major genes inducing resistance against a wide range of *P*. *teres* f. *teres* isolates [6, 11, 14, 19, 22–26, 30–34]. However, despite numerous studies conducted, the exact relationship among the various *P*. *teres* f. *teres* QTL and resistance genes remains uncertain as studies used different populations, isolates, and marker types [19].

The majority of QTL and genes conferring resistance to *P*. *teres* f. *teres* have been identified by bi-parental linkage mapping (LM, reviewed in [4]). Association mapping (AM) to detect *P*. *teres* f. *teres* resistance QTL was applied only in the study of Richards et al. [21]. Up to now, no nested association mapping (NAM) study has been performed to identify QTL linked to resistance to *P*. *teres* f. *teres*. The NAM concept is based on a multi-parental mapping design and was introduced as a genome-wide complex trait dissection strategy by Yu et al. [35]. NAM combines the advantages of conventional LM and AM strategies, namely the increased power of QTL detection and the increased allelic variation compared to bi-parental populations, allowing for an exceptional high mapping resolution [35–37]. Next to several studies based on the initial maize NAM population [35, 36, 38–45], NAM studies focusing on sorghum [46], wheat [47, 48], barley [49–53] and maize [54] highlight the power of this mapping approach.

Up to now, the world’s first barley NAM population introduced by Maurer et al. [49] named ‘Halle Exotic Barley 25’ (HEB-25) has not been used to identify QTL linked to biotic stress resistance. Thus, in this study the high genetic diversity present in HEB-25 and the high mapping power offered by NAM was used to achieve the five main objectives: I) to screen the HEB-25 population for resistance against *P*. *teres* f. *teres*; II) to identify HEB-25 lines showing high resistance suitable to be introduced in pre-breeding programs; III) to identify net blotch resistance QTL by NAM based on two resistance measures; IV) to compare QTL positions found in this study with those previously reported in literature, and V) to identify putative candidate genes underlying the identified resistance QTL.

## Material and methods

### Plant material

This study is based on the HEB-25 NAM population [49]. HEB-25 comprises 1,420 BC_1_S_3_ lines in 25 families originating from a cross of 25 highly diverse wild barley accessions (*Hordeum vulgare* ssp. *spontaneum* and *H*. *agriocrithon*) with the modern spring barley cultivar Barke (*Hordeum vulgare* ssp. *vulgare*). For more detailed information on population development see Maurer et al. [49]. Due to a loss of genotypes during field trials the analysis is based on 1,403 genotypes of the HEB-25 population.

### Field trials

Field trials were conducted at the Julius Kuehn-Institute, Federal Research Centre for Cultivated Plants, in Quedlinburg, Germany, in 2014 and 2015 using a special experimental design called summer hill trial design developed by König et al. [17]. Genotypes were sown in rows of so called hill-plots comprising 25 seeds each, with a spacing of 0.5 m between hills. Spreader strips of susceptible varieties (Candesse and Stamm 4046) were sown between hill-plot rows with a row to row spacing of 1.0 m (S1 File). Trials were laid out in a randomized incomplete block design with two replicates of 18 incomplete blocks each. A resistant standard (gene bank accession HHOR 10860) was integrated three times in each of the incomplete blocks. Net blotch (*P*. *teres* f. *teres*) infected barley straw was incorporated in the topsoil before sawing to serve as a source of infection and to ensure homogenous disease pressure. Infected barley plants were harvested at the end of the first year and the straw was used as infection material for the second year. Field trials were sown in the first half of August as König et al. [17] had shown that at that time growing conditions in Germany are more favorable for *P*. *teres* f. *teres* in comparison to *Rhynchosporium secalis*, which is often opposite in spring, thereby preventing reliable scoring of *P*. *teres* f. *teres* resistance.

### Phenotypic data

The percentage of infected leaf area (PILA), according to Moll et al. [55], and the reaction type (RT), applying the disease scale of Tekauz [56], were recorded at three consecutive dates, starting when disease symptoms were clearly visible in the susceptible spreader strips. A time period of two weeks between phenotyping dates was chosen to allow for a sufficient disease development. PILA data was used to calculate the area under the disease progress curve (AUDPC). AUDPC data was then used to calculate the average ordinate (AO) as a measure of infection severity:

$$AO=\frac{\sum_{i=1}^{N_{i-1}}\frac{\left(y_{i}+y_{i+1}\right)}{2}*(t_{i+1}-t_{i})}{tp}$$

where (*N*) is the total number of observations, disease level at the ith observation is coded by (*y*_*i*_), time at the ith observation is coded by (*t*_*i*_) and the trial period in days is coded by (*tp*).

### Statistical analysis

Phenotypic data analysis was performed using the software package SAS 9.4 (SAS Institute Inc., Cary, NC, USA) using *proc mixed*. Genotype, year, and genotype *x* year interaction were set as fixed. Design effects were set as random statement. Separate covariances were set for years to account for the difference in disease pressure between years. AO least-squares means (lsmeans) as well as RT lsmeans were used for subsequent NAM.

To estimate variance components to be used for the calculation of broad sense heritability, all model parameters were set as random. Broad sense heritability across years was calculated as:

$$h^{2}=\frac{V_{G}}{V_{G}+\frac{V_{GY}}{y}+\frac{V_{R}}{yr}}$$

where genotypic variance is coded by (*V*_*G*_), genotype *x* year variance is coded by (*V*_*GY*_), and residual variance is coded by (*V*_*R*_). The terms *y* and *r* indicate the number of years and replicates, respectively.

Pearson’s correlation coefficients were calculated with *proc corr*, using lsmeans per genotype as input.

### Nested association mapping

SNP genotyping was carried out using the barley Infinium iSelect 9K chip consisting of 7,864 SNPs [57]. SNPs showing >10% failure rate, >12.5% heterozygous calls, or being monomorphic over all 1,403 HEB lines were removed from the dataset. SNP filtering resulted in 5,715 informative SNPs used for NAM with an average genetic distance of 0.17cM and a maximum gap of 11.1cM between adjacent markers. Linkage disequilibrium (LD) across HEB-25 was calculated as r^2^ between all mapped SNPs, excluding heterozygous genotypes, with the software package TASSEL 5.0 [58]. LD decay across intra-chromosomal SNPs was displayed by plotting r^2^ between SNP pairs against their genetic distance. A second-degree smoothed loess curve was fitted in SAS with *proc loess*. The population-specific baseline r^2^ was defined as the 95^th^ percentile of the distribution of r^2^ for unlinked markers [59]. LD decay was defined as the distance at which the loess curve crosses the baseline. An identity-by-state approach was used to differentiate HEB genotypes. Parental genotype information enabled the identification of the exotic donor allele in each segregating HEB family. HEB lines showing a homozygous Barke genotype were assigned a value of 0, HEB lines showing a homozygous exotic genotype were assigned a value of 2, and heterozygous HEB lines were assigned a value of 1. Failed SNP calls were assigned a value using the mean imputation (MNI) approach [60]. For detailed information see Maurer et al. [49]. Assignment of SNPs to chromosomal positions was based on Maurer et al. [49].

NAM was performed using Model B of Liu et al. [61] verified to be best suited for genome-wide association studies (GWAS) based on family-structured populations [62] and successfully applied in previous HEB-25 studies [49–51]. Model B is a multiple regression model including, next to a quantitative SNP effect and a qualitative family effect, quantitative cofactors that correct for population stratification and genetic background noise [62]. Marker trait associations were estimated by stepwise forward-backward regression based on minimizing the Bayesian information criterion (BIC [63]) taking all informative SNPs into consideration. Analysis was carried out with SAS 9.4 applying the *proc glmselect* procedure. SNPs were allowed to enter or leave the model at each step until the BIC estimate was not reduced any further. SNPs included in the final model were defined to be significant.

To increase the robustness of identified marker trait associations, a five-fold cross-validation (CV) was performed. In total, 200 CV runs (40 times five-fold CV) were performed. For this, 200 subsets were extracted out of the full genotype set. Subsets included 80% of genotypes of the full population each, randomly selected per HEB family. The subsets were taken as training sets for the identification of significant marker trait associations and for estimation of additive effects. The remaining 20% of genotypes were used as the validation set. Subsequently, the count of each significant marker over all training sets was recorded and referred to as detection rate (DR). This value was taken as a measure of robustness of the marker trait association. Markers with a DR of >50% were defined as particularly robust and used to assign resistance QTL.

Additive effects for each SNP were extracted as regression coefficient of the respective SNP directly from the NAM model described above. To obtain final estimates, additive effects of significant markers were averaged across all runs. Likewise final R^2^ values for significant SNPs were obtained by averaging R^2^ values of significant markers across all cross-validation runs. This way, the R^2^ value can be interpreted as the percentage of variance explained by the investigated SNP marker. Furthermore, hotspots of marker trait associations were assigned to chromosome regions by determining the count and the mean additive effect of significant markers within 5cM.

A standard QTL interval of ±4cM around the markers with a DR >50% was defined, resembling the LD decay in HEB-25 (S2 File). In case the QTL was composed of more than one marker with a DR >50%, the marker showing the highest DR across all 200 cross-validation runs was defined as peak marker. QTL showing overlapping QTL intervals were combined to a single QTL interval.

To estimate the proportion of phenotypic variance explained by the full model, the unbiased estimator R^2^_adj_ [64] was calculated for each subset by simultaneously modeling all of the significant markers in the linear model described above.

To determine the predictive ability R^2^_pred_ of the full model for infection severity, the additive effects of markers estimated using the training sets were used to predict the phenotypic value of the remaining 20% of genotypes forming the validation sets [65]. Following Maurer et al. [50] R^2^_pred_ was defined to be the squared Pearson product-moment correlation between predicted and observed phenotypic values. Subsequently, R^2^_adj_ and R^2^_pred_ values were averaged over all 200 CV runs to obtain final estimates.

Additional to the detection of marker trait associations across families, parent-specific QTL effects were calculated following the approach of Maurer et al. [52]. In a first step, the peak marker (SNP with highest DR >50% across all 200 cross-validation runs) of each QTL was selected and placed central in a 26cM interval (resembling the mean introgression size in HEB-25) to look for significant SNPs in this region. Due to model limitations reported in Maurer et al. [52] population-wide QTL located within this interval were pooled into one single parent-specific QTL. Subsequently ‘Model-B’ SNP effect estimates of all markers within this interval were cumulated for each of the 25 donors, following $\sum_{i}^{n}{SNP\;(donor)}_{i}{*\alpha }_{i}$, where (*i*) iterates through all significant SNPs (*n*) in the respective QTL interval. *SNP* (*donor*)_*i*_ represents the quantitative IBS donor genotype (i. e. 0 vs. 2) of the ith significant SNP and *α*_*i*_ denotes the SNP effect estimate of this SNP obtained from ‘Model-B’. Since SNPs show different IBS segregation patterns across the donors of HEB families a different cumulated effect was obtained for each donor. This procedure was conducted within each of the 200 cross-validation runs. Subsequently, the mean effect across all cross-validation runs was calculated and taken as the final parent-specific QTL effect estimate.

### Comparison with previously identified QTL and analysis of identified QTL intervals

GrainGenes (https://wheat.pw.usda.gov/GG3/) and BARLEX (http://apex.ipk-gatersleben.de/apex/f?p=284:10) databases were searched to obtain marker sequence information on previously reported QTL for net blotch resistance. If available, the marker sequence information was used to check for overlap of net blotch resistance QTL identified in this study with those 23 studies reported before and cited in the introduction. Only those QTL from previous studies were taken into consideration, which were placed in similar chromosomal regions as our QTL. The BARLEYMAP pipeline [66] was used as a common reference. Using this pipeline, the peak marker as well as flanking markers for known net blotch resistance QTL and markers identified in this study showing a DR >50% were blasted against the POPSEQ map [67] and the barley physical map [68]. Markers with a DR >50% identified in this study and located in a genetic distance of less than 4cM (resembling the LD decay in HEB-25, see S2 File) to markers of known resistance QTL were defined as potentially corresponding to previously reported resistance QTL. In addition, previously reported QTL for which no marker information could be obtained were compared to QTL detected in this study based on information given in the respective publication.

In addition, the BARLEYMAP pipeline [66] was used to identify potential candidate genes underlying the particularly robust QTL of this study by aligning the associated markers showing a DR >50% against the barley physical map [68] and the POPSEQ map [67]. The gene search was extended to an interval of ±4cM around markers with a DR >50% to account for the LD decay in HEB-25. Gene ontology (GO) terms defining defence response (0006952, 0050832), apoptotic process (0006915), peroxidase activity (0004601), response to (oxidative) stress (0006979, 0006950), ATP binding (0005524), nucleotide binding (0000166), protein binding (0005515), transporter activity (0005215), protein kinase activity (004672) were used to validate genes involved in resistance reactions [69]. Furthermore, GO terms defining reactions potentially involved, e.g. catalase activity, chitinase activity, cell wall, peroxisome, cell wall modification, and defence response to fungi, were considered (S8 File).

## Results

### Phenotypic data

**Fig 1:**
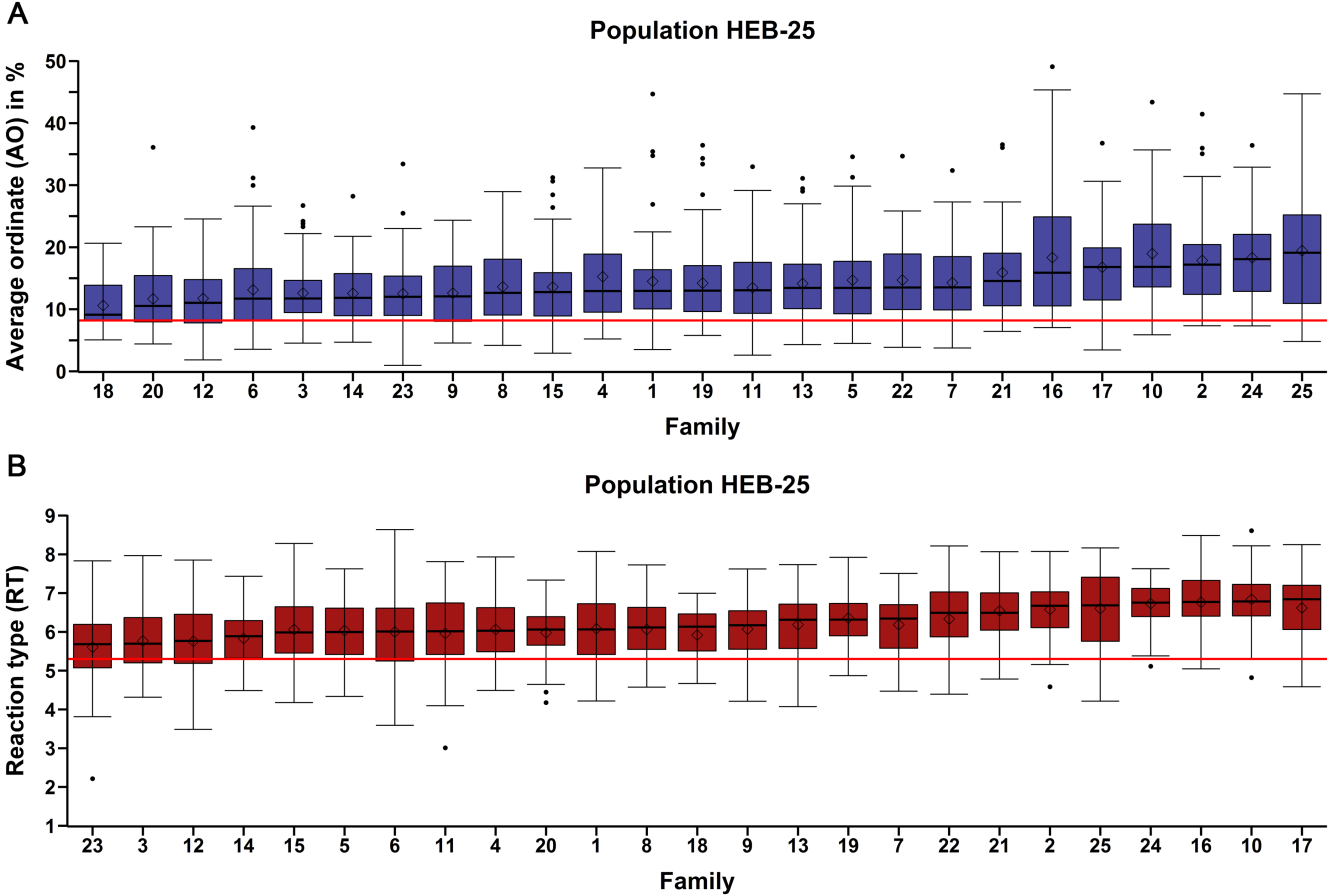
Box-whisker plots per HEB family indicating the variation in genotype responses to net blotch infection. **(A)** average ordinate (AO) and **(B)** reaction type (RT). The y-axis shows the data for each trait; the x-axis depicts the 25 families of HEB-25 (1–25) sorted by ascending median. The red line depicts the value of the resistant check for the respective trait.

In both years the use of the summer hill trial design resulted in an elevated disease pressure across the whole field with spreader strips showing an AO close to 60%. The experimental conditions allowed for an optimal differentiation of the degree of *P*. *teres* f. *teres* resistance between genotypes. A large diversity in *P*. *teres* f. *teres* resistance of genotypes was observed for both traits studied with a highly significant variation (p <0.0001; Tukey-test) between as well as within families of the HEB-25 population (Fig 1A and 1B; S3 File).

A wide range of genotype responses to *P*. *teres* f. *teres* infection was observed in HEB-25 with a range of 48.2% for AO (0.95–49.1%) and 6.4 for RT (2.21–8.64), respectively (Table 1). Notably, several genotypes of the HEB-25 population showed a higher degree of resistance than the resistant check included in field trials (Fig 1A and 1B). The top 1% of all genotypes regarding *P*. *teres* f. *teres* resistance showed a mean AO value of 2.9% and a mean RT value of 3.8 (S3 File). The frequency distributions for both traits were slightly right skewed (S4 File). Because of the HEB-25 population design, the population means are close to the recurrent parent Barke (Table 1). Barke showed a high degree of susceptibility compared to the majority of wild donor parents. Only the wild donor of family 24 (*Hordeum vulgare* ssp. *agriocrithon*), originating from Tibet, China, showed a higher *P*. *teres* f. *teres* susceptibility than Barke (S5 File).

**Table 1:** Descriptive statistics for two-year least-squares means (lsmeans) and heritability.

Trait^a^	N^b^	Mean Barke^c^	Mean HEB-25^d^	Min^e^	Max^f^	SE_+/-_^g^	CV^h^	h^2^^i^
AO	1403	13.91	14.65	0.95	49.1	0.19	0.48	0.62
RT	1403	6.64	6.20	2.21	8.64	0.02	0.14	0.65

^a^Average ordinate (AO), reaction type (RT).

^b^Number of genotypes analysed.

^c^Two-year lsmeans of commom parent Barke.

^d^Two-year lsmeans of the HEB-25 population.

^e^Minimum.

^f^Maximum.

^g^Standard error.

^h^Coefficient of variation.

^i^Broad-sense heritability.

Two-year broad sense heritability was calculated to be h^2^ = 0.62 for AO and h^2^ = 0.65 for RT, respectively (Table 1). High correlations (Pearson’s correlation coefficients; p <0.0001) were observed between the two resistance measures AO and RT with r = 0.86 and r = 0.76 for HEB-25 parents and for the HEB-25 population, respectively (S5 File).

### Nested association mapping

**Fig 2:**
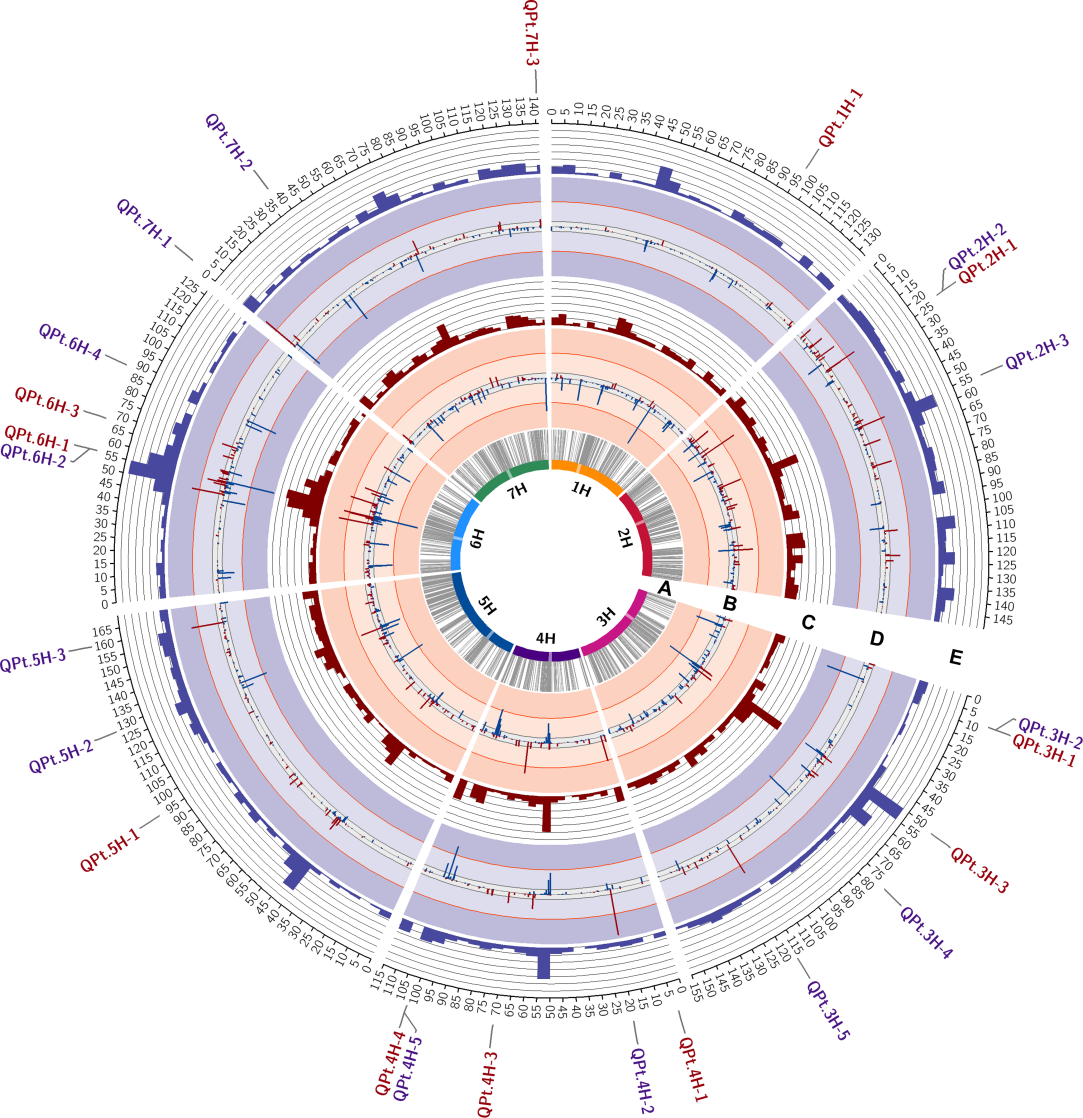
Circos plot indicating QTL involved in net blotch resistance, i.e. average ordinate (AO) and reaction type (RT). The barley chromosomes are arranged as coloured bars forming the most inner circle. Centromere regions are highlighted as transparent boxes. **(A)** Grey connector lines represent the genetic position of the 5,715 informative SNPs on the chromosomes with cM positions based on Maurer et al. [49] given on the scale on the outside of circle E. **(B)** Marker trait associations calculated for reaction type (RT). Bars identify the position and detection rate (DR, height of bars) of significant marker trait associations. Bars in blue, pointing inwards, indicate a population wide trait-decreasing effect exerted by the exotic allele, whereas bars in red, pointing outwards, indicate a population wide trait-increasing effect exerted by the exotic allele. The grey and orange lines depict the DR threshold of 10% and 50% across 200 cross-validation runs. **(C)** Count of significant marker trait associations within 5cM intervals for the NAM study based on RT data. **(D)** Marker trait associations calculated for average ordinate (AO). Graphical representation as described under (A). **(E)** Count of significant marker trait associations within 5cM intervals for the NAM study based on AO data. The position of particularly robust QTL with DR >50% are indicated on the scale outside of circle E. QTL detected based on RT are shown in red, whereas QTL detected based on AO are shown in purple.

NAM was performed for the two traits AO and RT, resulting in the identification of a high number of significant marker trait associations (Fig 2; S6 File). Most marker trait associations showed a DR below 50% across the 200 cross-validation runs. However, 11 and 13 particularly robust QTL being composed of one or more markers with a DR above 50% were identified for RT and AO, respectively (Table 2). Particularly robust marker trait associations were identified on all chromosomes except chromosome 1H in case of AO and on all chromosomes for RT. The QTL showing the peak marker with the highest DR (i_SCRI_RS_186193) is located in the centromeric region of chromosome 6H for both traits evaluated (Fig 2; Table 2). This QTL is composed of three SNPs with DR >50% in case of RT and two SNPs in case of AO. In both cases the peak marker showed a negative cross-validated mean effect, i.e. an increase of resistance in the presence of the wild allele compared to the Barke control allele. In general, this chromosome region showed the highest abundance of significant marker trait associations (Fig 2).

**Table 2:** Robust net blotch resistance QTL (DR >50%) detected in the two NAM studies.

QTL	Chr^a^	Markers with DR >50%^b^	Position of peak marker (cM)^c^	DR in 200 CV runs (%)^d^	CV mean R^2^ (%)^e^	CV mean allele effect^f^	Corresponding net blotch QTL/genes^g^
**Reaction type (RT)**							
QPt.1H-1	1H	i_11_10357	95.9	85.5	2.33	-0.28	
QPt.2H-1	2H	i_BK_15	23	78	9.23	+0.50	QRpts2Sa^1^ QNFNBAPR.Ar/F-2H^1^ QTL_Steffenson^1^
QPt.3H-1	3H	i_11_10112	8.5	54.5	0.87	-0.22	QTL_UHs_-3H-1^3^
QPt.3H-3	3H	i_11_10966	51.6	73.5	8.64	-0.98	QTL_UHs_-3H-2^3^ QTL_Liu^5^
QPt.4H-1	4H	i_SCRI_RS_206744	3.5	51.5	1.04	+0.21	QRptts-4HS^7^
QPt.4H-3	4H	i_SCRI_RS_175327	70.3	63.5	0.85	+0.51	QRpts4^1^ Rpt-4H-5–7^1^
QPt.4H-4	4H	i_SCRI_RS_167808	101.7	53.5	6.64	-0.52	QNFNBAPR.W/AI-4H^1^ QNFNBAPR.AI/S-4Hb^1^
QPt.5H-1	5H	i_11_10834	94.7	58	1.63	-0.29	QTL_UH_-5H-1^2^ QRptts-5HL.2^7^
QPt.6H-1	6H	**i_SCRI_RS_186193** i_11_10013 i_SCRI_RS_239642	55.7	90 69 61.5	0.08	-0.68	Rpt5^1^* Rpt-r/-k^1^* Rpt-Nomini/-CIho2291^4^* Spt1^6^* SPN1^5^*, 6H QTL^8^
QPt.6H-3	6H	i_SCRI_RS_157316	67.6	56.5	1.37	+0.36	QTL_Liu^5^
QPt.7H-3	7H	i_SCRI_RS_123211	140.7	66	0.10	-0.21	
**Average ordinate (AO)**							
QPt.2H-2	2H	**i_BK_12** i_BK_13	23	68 51	14.88	+4.74	QRpts2Sa^1^ QNFNBAPR.Ar/F-2H^1^ QTL_Steffenson^1^
QPt.2H-3	2H	i_SCRI_RS_13639	55.55	58	0.07	+4.05	QTL_Cakir^1^ QNFNBAPR.W/AI-2H^1^ QRpts2Sb^1^
QPt.3H-2	3H	i_11_10112	8.5	78.5	0.78	-1.59	QTL_UHs_-3H-1^3^
QPt.3H-4	3H	i_12_10583	77.4	55.5	0.04	-2.86	QRpts3La^1^ QNFNBAPR.W/AI-3H^1^ QNFNBAPR.AI/S-3H^1^
QPt.3H-5	3H	i_SCRI_RS_146197	117	67	0.12	+5.65	QRpts3L^1^ QNFNBAPR.AI/S-3H^1^ QNFNBAPR.W/AI-3H^1^ QTL_Liu^5^ QTL_PHs_-3H^3^
QPt.4H-2	4H	i_12_30150	19.9	93.5	0.36	+1.92	
QPt.4H-5	4H	i_SCRI_RS_167808	101.7	68	6.50	-3.67	QNFNBAPR.W/AI-4H^1^ QNFNBAPR.AI/S-4Hb^1^
QPt.5H-2	5H	i_SCRI_RS_228463	128.2	56	1.50	-2.51	QTL_PH_-5H-3^2^ QRptts5^1^
QPt.5H-3	5H	i_11_21138	159.8	64.5	0.41	+1.72	
QPt.6H-2	6H	**i_SCRI_RS_186193** i_11_10013	55.7	98 65.5	0.39	-5.99	Rpt5^1^* Rpt-r/-k^1^* Rpt-Nomini/-CIho2291^4^* Spt1^6^* SPN1^5^*, 6H QTL^8^
QPt.6H-4	6H	i_SCRI_RS_7640	87.9	61.5	0.04	-2.24	
QPt.7H-1	7H	**i_SCRI_RS_200895** i_SCRI_RS_156237	0.6	77 59.5	3.65	+9.64	QNFNBAPR.AI/S-7Ha^1^ QTL_PH_-7H^2^
QPt.7H-2	7H	i_SCRI_RS_179937	37.6	60	1.54	-1.97	

^a^Barley chromosome on which the QTL is located.

^b^SNP name of markers with a detection rate (DR) >50% associated with the QTL. In case the QTL is composed of several markers, the QTL peak marker is shown in bold letters.

^c^Position of the QTL peak marker showing highest DR based on Maurer et al. [49].

^d^Detection rate of the QTL peak marker in 200 cross-validation runs in percent.

^e^Mean percentage of phenotypic variance explained by the QTL peak marker based on 200 cross-validation runs.

^f^Population-wide mean effect of the QTL peak marker based on 200 cross-validation runs. Positive and negative signs indicate a trait-increasing and trait-decreasing effect of the wild allele compared to the Barke control allele, respectively.

^g^Previously reported net blotch resistance QTL/genes located within the range of LD decay around the QTL marker with DR >50% identified in this study (^1^reviewed in [4], ^2^[17], ^3^[18], ^4^[25], ^5^[19], ^6^[26], ^7^[21], ^8^[70]

*for QTL defining the same position see [4, 20, 21]).

A considerable variation in the wild allele effect estimates of adjacent markers was observed with an increase or decrease in trait values compared to the Barke control allele (Fig 2). Notably, the majority of detected QTL are composed of marker trait associations exhibiting opposed wild allele effect estimates (S6 File). For both traits QTL peak markers exhibited the same effect direction as the mean QTL estimate in all but one case, but differed in effect size (S7 File). Thus, NAM showed that QTL and peak marker effect are not necessarily identical. Across the whole population particularly robust QTL peak markers showed predominantly small to intermediate wild allele effect estimates and low R^2^ values. Wild allele effects ranged from -5.99 to 9.64 in case of AO and from -0.98 to 0.51 in case of RT. The peak markers of QPt.7H-1 and QPt.3H-3 showed the highest effect estimate for AO and RT, respectively (Table 2). R^2^ values ranged from 0.04 to 14.88% explained variance in case of AO and from 0.08 to 9.23% in case of RT (Table 2). The peak markers of QPt.2H-2 and QPt.2H-1 showed the highest R^2^ value for AO and RT, respectively (Table 2).

Parent-specific QTL effects were calculated to obtain an effect estimate resembling the combined effect of all family specific markers the QTL is composed of. Due to previously mentioned model limitations (see Material and methods) QTL QPt.6H-1 and QPt.6H-3 were combined to one single parent-specific QTL (QPt.6H-1/3). Estimation of parent-specific QTL effects revealed a high variation in effect sizes of the wild allele among HEB families (S6 File). In most cases even the effect direction varied. For each trait five QTL (AO: QPt.2H-2, QPt.3H-4, QPt.4H-5, QPt.6H-4, QPt.7H-2; RT: QPt.1H-1, QPt.2H-1, QPt.3H-1, QPt.4H-3, QPt.4H-4) showed the same wild allele effect direction across all families (S6 File). No family showed trait-reducing effects at all parent-specific QTL in case of both traits. The maximum count of parent-specific QTL showing a trait-reducing effect of the wild allele were nine for AO (family F15) and seven for RT (family F23) (S6 File). For AO the three families F07 (-5.38%), F12 (-4.67%), and F15 (-3.82%), and for RT families F12 (-1.70), F07 (-1.34), and F23 (-1.25) showed the highest trait-reducing effects for wild type alleles summed up over all parent-specific QTL (S6 File).

Between the two NAM studies a considerable overlap was observed. Of the particularly robust 11 and 13 QTL peak markers identified for AO and RT, four peak markers mapped to the same or to a nearby position (Fig 2). In three cases, QTL even shared the same peak marker (Table 2). However, trait-specific QTL existed likewise. Hotspots of marker trait associations, defined by a high number of significant markers in the respective 5cM interval, corresponded well with QTL peak marker positions in most cases. Similar to the observations in case of QTL peak markers, considerable overlap between trait hotspot regions of the two traits analysed existed (Fig 2).

The mean percentage of phenotypic variance explained by the full model (R^2^_adj_) was calculated to be 68.9% for AO and 72.0% for RT (Table 3). Notably, for both traits a considerable fraction of the phenotypic variance was explained by the identified particularly robust QTL peak markers (Table 2). The predictive ability (R^2^_pred_) of the full model for infection severity was calculated to be 42.1% for AO and 43.3% for RT (Table 3).

**Table 3:** Number of QTL and total phenotypic variance explained.

Trait^a^	QTL^b^	R^2^_adj_ (%)^c^	R^2^_pred_ (%)^d^
AO	13	68.9	42.1
RT	11	72.0	43.3

^a^Average ordinate (AO), reaction type (RT).

^b^Number of QTL define for the respective trait.

^c^Mean phenotypic variance explained by the full NAM model.

^d^Mean ability to predict infection severity of independent genotypes.

### Comparison with previously identified QTL

Comparison of net blotch resistance QTL identified in this study with those already reported in literature revealed that the majority of identified QTL mapped to chromosome regions known to be linked to net blotch resistance. In case of RT nine out of 11 QTL showed overlap with QTL intervals of previously reported resistance QTL or genes, whereas this was true for nine out of 13 for AO (Table 2). In detail, based on available data no overlap was found for QPt.1H-1, QPt.4H-2, QPt.5H-3, QPt.6H-4, QPt.7H-2, and QPt.7H-3. Out of these QTL, peak markers of QPt.1H-1, QPt.6H-4, QPt.7H-2, and QPt.7H-3 revealed negative CV mean effects (Table 2) indicating the existence of wild barley alleles conferring net blotch resistance. The alignment of SNPs with DR >50% against the physical barley map by means of the BARLEYMAP pipeline resulted in the identification of a number of genes related to plant defence in the respective QTL intervals. In particular, leucine-rich repeat, NB-ARC, and Serine/threonine-protein kinase-like domain genes were found at high frequency. Details are given in S8 File.

In addition, QTL analysis revealed that peak markers of QPt.2H-1 and QPt.2H-2 are SNPs of the barley pseudo-response regulator gene *Ppd-H1*. Based on this finding, other QTL identified in our study were compared to flowering time QTL identified in an earlier HEB-25 study by Maurer et al. [49]. In addition to QPt.2H-1 and QPt.2H-2, overlap of QTL QPt.2H-3, QPt.5H-2, and QPt.7H-2 with flowering QTL QFt.HEB25-2c, QFt.HEB25-5d, and QFt.HEB25-7a of Maurer et al. [49] was observed. Furthermore, QPt.2H-3, QPt.5H-2, and QPt.7H-2 each showed to include MLOC numbers present in the corresponding flowering time QTL identified in the study by Maurer et al. [49] and identified to correspond to flowering time related genes *HvCEN*, *Vrn-H1*, and *Vrn-H3*, respectively.

## Discussion

The high variation in *P*. *teres* f. *teres* infection severity observed in field trials clearly reflects the high genetic diversity present within the HEB-25 population, which is in line with findings of previous HEB-25 NAM studies focusing on developmental traits [49, 50] and salinity tolerance [51]. The presence of significant differences not only between families but also within families demonstrates the high suitability of HEB-25 to identify population-wide as well as parent-specific QTL for *P*. *teres* f. *teres* resistance (Fig 1A and 1B; S3 File).

Phenotypic results of this study show that the high variation to net blotch resistance can be attributed to combined effects of a diverse set of predominantly highly resistant wild donor parents of HEB-25 and a relatively susceptible recurrent parent Barke. HEB-25 lines identified to possess a higher degree of resistance than the highly resistant check line included in field trials represent suitable candidates for pre-breeding programs (S3 File). Results of earlier studies by Maurer et al. [49, 50] may be considered to select those net blotch resistance conferring HEB lines that combine high *P*. *teres* f. *teres* resistance with favorable yield related parameters. Advantageous is that integration of HEB-25 lines into pre-breeding programs will be faster to achieve than in case of the integration of wild accessions since a backcrossing step with cultivar Barke was already performed during population development.

The summer-hill trial design developed by König et al. [17] proved to be highly effective, allowing for a clear differentiation of genotype responses, thereby laying the basis for successful QTL identification with NAM. The high correlation between the two infection severity measures applied in this study and the relatively high heritabilities found prove that both measures allow a reliable scoring of genotypic resistance (Table 1; S5 File).

The occurrence of opposed wild allele effect estimates of closely linked markers in this study was also observed in previous HEB-25 studies by Maurer et al. [49, 50, 52] and likely arises from a combination of factors. Firstly, not all SNPs segregate in all genotypes and therefore, markers are likely to reflect only the mean wild allele effect of a fraction of the full population. As a result, closely linked markers segregating in different sets of genotypes of the full population can show opposed effect estimates because of different mean resistance levels of the two sets. Phenotypic results revealed that families differ in their mean resistance level (Fig 1A and 1B). Therefore, it can be assumed that strongly differing sets are likely to be linked to different families and, thus, opposed effect estimates of closely linked SNPs can be caused by parent-specific alleles. Secondly, the presence of closely linked SNPs showing opposed wild allele effects can be caused by closely linked alleles with contrasting effects on *P*. *teres* f. *teres* resistance. A good example is the centromeric region of chromosome 6H that is known to harbor a number of closely linked *P*. *teres* f. *teres* resistance genes of which some are assumed to be in repulsion [4, 19, 25, 26, 70]. Therefore, we assume that the high number of closely linked markers with opposed wild allele effect estimates in the centromeric region of chromosome 6H identified in this study is likely to be partially caused by this complex cluster of resistance related genes.

In this study a rather stringent threshold for the acceptance of marker trait associations was defined. Therefore, minor QTL not passing this threshold but still influencing genotype response to *P*. *teres* f. *teres* are not considered. Defining a less stringent DR threshold of 10%, as applied in the study of Maurer et al. [50], would have resulted in a considerable higher number of QTL (Fig 2; S6 File). Nevertheless, hotspots of marker trait associations identified in this study may be used to narrow down regions potentially harboring minor QTL involved in the resistance response of genotypes to *P*. *teres* f. *teres*. However, when analyzing the hotspot information it has to be taken into account that in centromeric regions the number of markers is generally high and, therefore, centromeric regions should be interpreted with caution (Fig 2). The detection of QTL despite low estimates across the whole population is a strong proof of the power of the NAM strategy in general and in particular the suitability and precision of the NAM model applied in this study (Fig 2; S6 File). The mean phenotypic variance explained by the full model and the calculated mean ability to predict the degree of infection of independent genotypes further supports the suitability of the applied model (Table 3).

The high number of QTL linked to net blotch resistance detected in this study, the small CV mean effect estimates as well as the low percentage of phenotypic variance explained by the majority of QTL peak markers indicate a complex inheritance of adult plant *P*. *teres* f. *teres* resistance (Table 2). This supports the conclusion drawn by Liu et al. [4] of a highly complex *P*. *teres* f. *teres*–barley interaction. Results of this study are comparable to previous NAM studies focusing on leaf blight in maize [38, 41] that identified variation in resistance to be a result of the accumulation of numerous small effect loci with additive effects. Likewise, NAM studies focusing on rust fungi of wheat [47, 48] resulted in the identification of a high number of QTL with predominantly small additive effect estimates. In addition, results of this study are comparable to the association study of Tamang et al. [71] focusing on resistance to the spot form of net blotch (*P*. *teres* f. *maculata*) and the association study of Richards et al. [21] focusing on seedling resistance to *P*. *teres* f. *teres*. The authors identified a high number of markers associated with resistance to *P*. *teres* f. *maculata* and *P*. *teres* f. *teres*, respectively, nearly all explaining only a low percentage of phenotypic variance.

Next to being the result of complex inheritance of *P*. *teres* f. *teres* resistance, small population-wide effects of QTL peak markers may also be attributed to the presence of alleles with differing effects on *P*. *teres* f. *teres* resistance. Namely, in case only a limited number of HEB-25 lines of the full population show a strong allele effect on resistance or contrasting allele effects among the 25 HEB donor parents exist at a marker position.

The importance of considering the influence of differing allele effects in HEB-25 on estimating a population-wide QTL peak marker effect is supported by results of the parent-specific QTL effect calculation (S6 File). An extreme example is the QTL QPt.7H-1. In this case, the high population-wide effect of the wild allele observed for the peak marker (wild barley allele effect on AO = +9.64) seems to be mainly caused by the strong effect of an allele or allele combination derived from the donor parent of HEB family F16 (wild barley parent-specific allele effect on AO in family F16 = +9.16). Comparable to this study, strongly varying parent-specific allele effects of QTL were observed likewise in the NAM studies of Bajgain et al. [47] and Li et al. [48] focusing on the identification of QTL conferring resistance to rust pathogens of wheat. Therefore, especially studies focusing on detailed analysis of specific QTL or the integration of net blotch resistance alleles in modern barley cultivars should use the parent-specific QTL effect information given in this study to select a resistance-carrying HEB line derived from the HEB family in which the estimated favorable QTL effect is maximized. Not including parent-specific QTL effect estimates in the selection decision may result in missing alleles whose strong favorable effect is masked by a high number of parent-specific alleles with an opposed effect on *P*. *teres* f. *teres* resistance (S6 File). However, in this regard it needs to be mentioned that parent-specific QTL effect estimates are likely to be slightly overestimated as each family comprises only a relatively small number of HEB-25 lines [52]. Thus, selection decisions should be based on a combined evaluation of population-wide and parent-specific estimates of the wild allele effect.

Several QTL identified in this study are located at chromosome positions not yet reported to be linked to *P*. *teres* f. *teres* resistance (Table 2). At the same time, QTL were identified that overlap with previously described *P*. *teres* f. *teres* resistance QTL. This fact is a strong proof of the reliability of the identified marker trait associations. NAM results are further supported by the fact that several QTL regions were independently identified by AO and RT (Fig 2; Table 2).

Out of the QTL that show no overlap some are located in the vicinity of previously reported *P*. *teres* f. *teres* resistance QTL. This is the case for QPt.1H-1 located in the vicinity of a QTL identified by Liu et al. [19], QPt.7H-2 located close to QTL QTL_UHs_-7H identified by König et al. [18], and QPt.7H-3 located in the vicinity of QTL QNFNBAPR.Al/S-7Hb identified by Lehmensiek et al. [5]. Furthermore, QPt.5H-3 is located in the region of a meta-QTL identified by Schweizer and Stein [72] effective against several fungal barley pathogens.

It has to be considered that previously reported *P*. *teres* f. *teres* resistance QTL were identified by the use of different isolates under different environmental conditions and mostly in seedling tests. Only QPt.5H-1, QPt.5H-2, and QPt.7H-1 showing overlap with QTL identified by König et al. [17] were identified under similar experimental conditions. Therefore, QTL identified in this study showing overlap with previously reported *P*. *teres* f. *teres* QTL should still be considered as distinct QTL until a test for allelism has been conducted.

Most of the identified particularly robust net blotch resistance QTL showed to be restricted to either AO or RT. These trait-specific QTL showed to be caused partly by the fact that for one trait the markers did not cross the defined DR threshold and thus, were not considered in this study, whereas for the other trait the markers crossed the threshold and were considered (Fig 2). In this case, for both traits DR peaks of markers were observed at the same or very close by positions and a less stringent threshold for the acceptance of marker trait associations (e. g. >10%, used by Maurer et al. [50]) would have resulted in the detection of the QTL based on both traits (Fig 2; S6 File). Furthermore, trait-specific QTL may be caused by the fact that the infection severity measure RT is less influenced by the degree of infection pressure, as a RT score indicative for susceptibility can be observed at a time point at which the fungus covers only a small percentage of the leaf (low AO value). Delaying the last phenotyping date, thus giving the fungus more time to spread across the leaf could have resulted in the detection of QTL regions based on both traits. Next to this study no other studies have been performed comparing AO and RT on the QTL level. Further research is needed to identify the underlying cause of these trait specific QTL.

The information given in this paper regarding genes located in a QTL region may assist in identifying the underlying genetic causes of a QTL effect (S8 File). The presence of leucine-rich repeat, NB-ARC, and Serine/threonine-protein kinase-like genes in the QTL intervals at high frequency is in agreement with findings of previous studies indicating an important role of those gene families in the necrotrophic effector triggered reaction to *P*. *teres* f. *teres* infection [19, 26]. Members of these gene families were also identified in other QTL studies focusing on necrotrophic and hemibiotrophic fungi [38, 41, 73]. The identification of various putative candidate genes by GO-term analysis may be viewed as a valuable source for subsequent studies focusing on the genetic basis of the *P*. *teres* f. *teres*–barley interaction (S8 File).

The overlap of QTL identified in this study with QTL identified to be linked to flowering time related genes [49, 50] points towards the involvement of the flowering time pathway in the resistance reaction to *P*. *teres* f. *teres*. Studies on *Arabidopsis thaliana* [74, 75] showed that QTL associated with resistance to the hemibiotrophic fungal pathogen *Verticillium* spp. mapped close to known flowering time genes and that the fungus influenced plant development. Association of flowering time with resistance to a necrothropic fungus has also been described in a study by Lyons et al. [76]. In this study a positive correlation between late flowering and resistance to *Fusarium oxysporum* in *A*. *thaliana* accessions was identified and the involvement of the photoperiodic pathway regulator GIGANTEA was shown. Furthermore, a negative correlation between days to anthesis and resistance to the hemibiotrophic maize pathogen *Exserohilum turcicum* has been identified [38, 77].

Detailed analysis of the identified overlap of QTL of this study with flowering time related QTL identified by Maurer et al. [49, 50] strongly points towards a negative correlation between flowering time and infection severity. Maurer et al. [49, 50] identified the wild alleles of *Ppd-H1* and *HvCEN* to cause early flowering, and, in contrast, the wild alleles of *Vrn-H1* and *Vrn-H3* to induce late flowering. In this study a resistance-decreasing effect of the wild allele was identified for peak markers of QTL overlapping with the *Ppd-H1* and *HvCEN* QTL, and a resistance-increasing effect for peak markers of QTL overlapping with the *Vrn-H1* and *Vrn-H3* QTL. Nevertheless, based on our study only a comparison of QTL localisation and QTL effects was possible. Further studies including phenotypic data and trials conducted during the standard growing period are required for final assessment.

The results of this study provide valuable information not only for fundamental studies focusing on elucidating the complex *P*. *teres* f. *teres*–barley interaction, but also for improving net blotch resistance and biodiversity of modern elite barley cultivars. In future, a better understanding of the allelic diversity present at net blotch resistance QTL in HEB-25 will be achieved, after an ongoing exome capture effort will result in detailed information on sequence diversity between 26 parental alleles at each known gene of a QTL region. This way, it is expected to achieve a clearer estimate of haplotype-based allele effects in HEB-25 and to foster the identification and selection of wild barley alleles, which increase net blotch resistance in barley.

## Supporting information

S1 File Summer hill trial design at an early and a later developmental stage. (PDF)

S2 File LD decay of intra-chromosomal markers across HEB-25. (PDF)

S3 File Net blotch least squares means (lsmeans) averaged across two years for average ordinate (AO) and reaction type (RT). (XLSX)

S4 File Frequency distribution of two-year lsmeans for trait average ordinate (AO) and reaction type (RT). (PDF)

S5 File Correlation between trait average ordinate (AO) and reaction type (RT) based on two-year lsmeans of HEB-25 parents and all HEB-25 lines. (PDF)

S6 File GWAS and cross validation results on net blotch measures of average ordinate (AO) and reaction type (RT). (XLSX)

S7 File Estimates of mean wild allele QTL effects and donor-specific QTL effects for net blotch measures of average ordinate (AO) and reaction type (RT). (XLSX)

S8 File Alignment of SNPs (DR >50%) with known genes based on BARLEYMAP. (XLSX)
