# Supplementary figures and images for: A nested association mapping population identifies multiple small effect QTL conferring resistance against net blotch (Pyrenophora teres f. teres) in wild barley
Source: PLoS One. 2017 Oct 26;12(10):e0186803. doi: 10.1371/journal.pone.0186803 (PMC5658061; doi:10.1371/journal.pone.0186803)

## Supplementary File 1

**A**

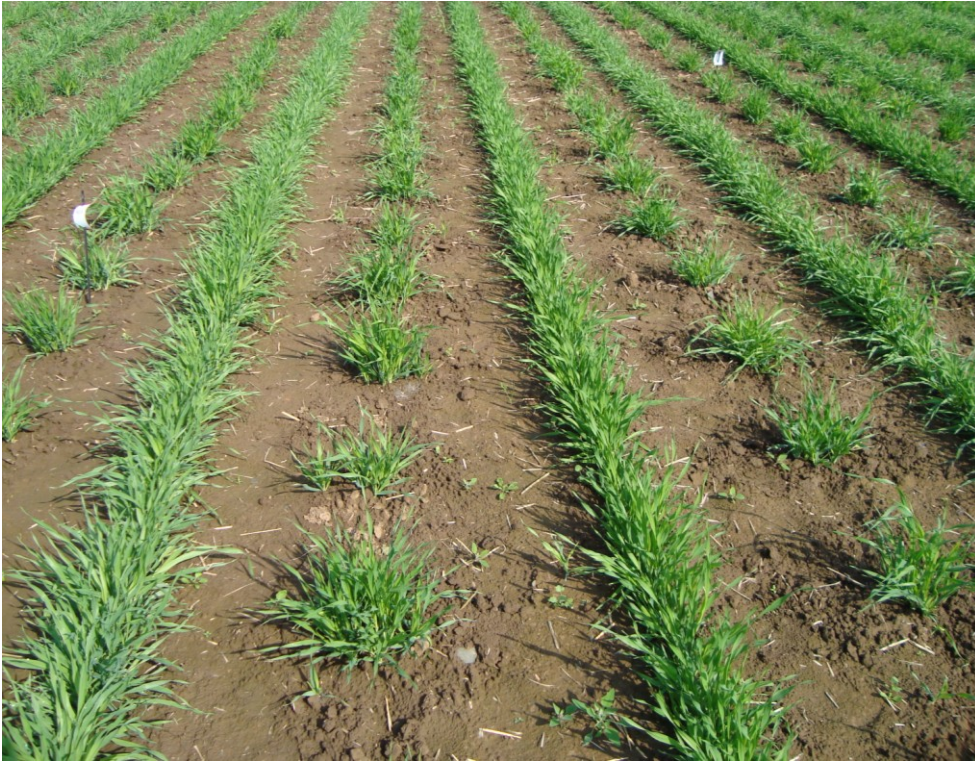

**B**

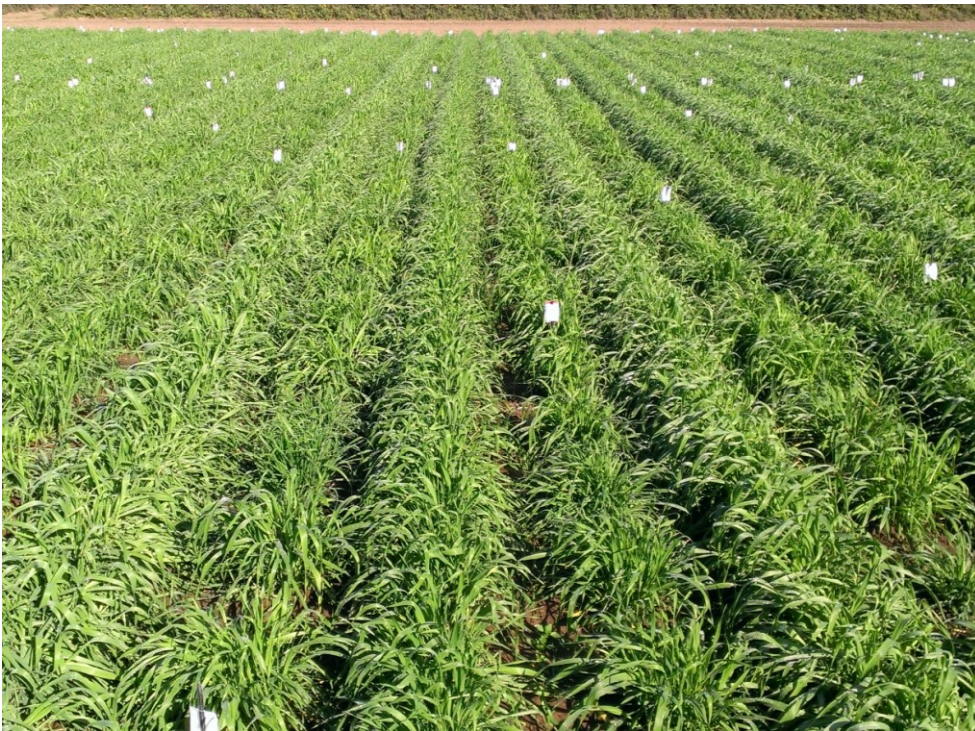

Summer hill trial design at an early (**A**) and a later (**B**) developmental stage.

Supplement: S1 File — (PDF) [file pone.0186803.s001.PDF]
