# Supplementary material for: A nested association mapping population identifies multiple small effect QTL conferring resistance against net blotch (Pyrenophora teres f. teres) in wild barley
Source: PLoS One. 2017 Oct 26;12(10):e0186803. doi: 10.1371/journal.pone.0186803 (PMC5658061; doi:10.1371/journal.pone.0186803)
Supplement: S2 File — (PDF) [file pone.0186803.s002.PDF]

Supplementary File 2

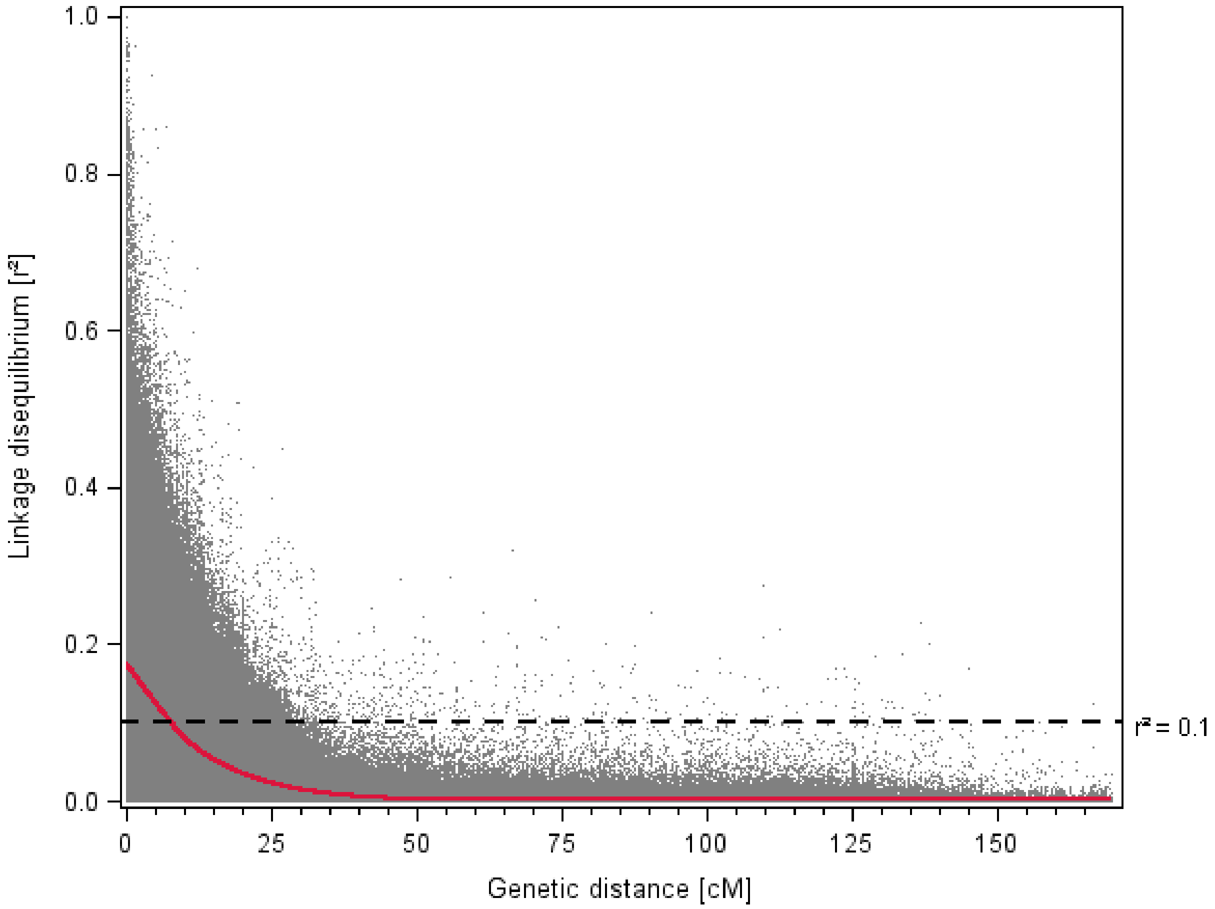

LD decay of intra-chromosomal markers across HEB-25. The red curve and the dashed black line indicate the second degree loess fit and the threshold of LD, based on the 95th percentile of inter-chromosomal SNPs, respectively. LD decay, defined as the distance at which the loess curve crosses the threshold, is 7.85.
