# Supplementary material for: A nested association mapping population identifies multiple small effect QTL conferring resistance against net blotch (Pyrenophora teres f. teres) in wild barley
Source: PLoS One. 2017 Oct 26;12(10):e0186803. doi: 10.1371/journal.pone.0186803 (PMC5658061; doi:10.1371/journal.pone.0186803)
Supplement: S4 File — (PDF) [file pone.0186803.s004.PDF]

Supplementary File 4

A

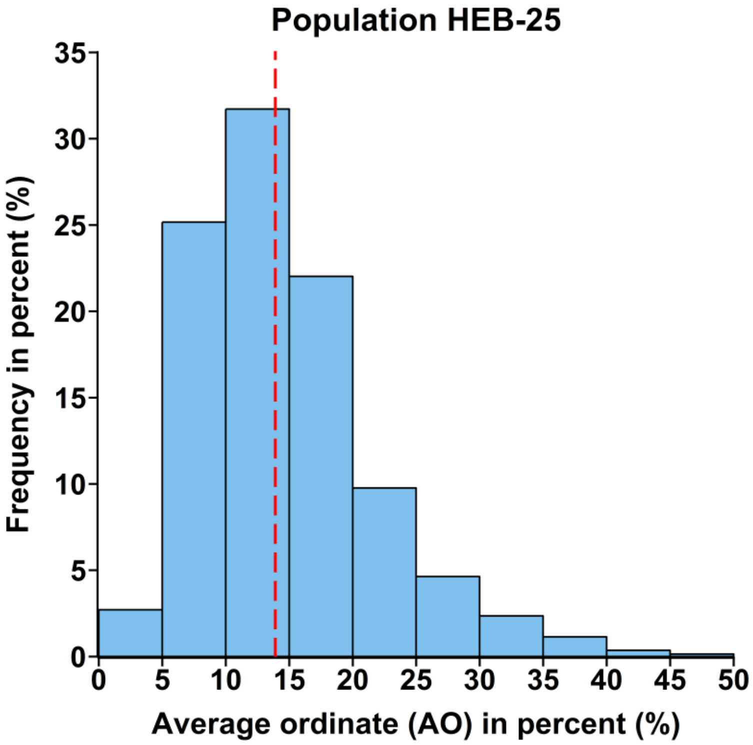

B

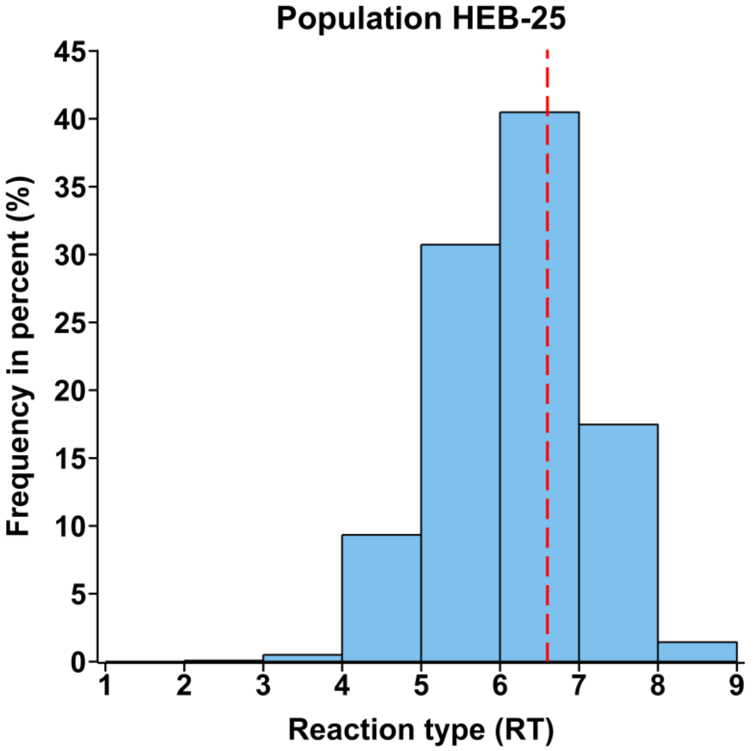

Frequency distribution of two-year lsmeans for **(A)** trait average ordinate (AO), and **(B)** trait reaction type (RT) on the bottom. The dotted red line depicts the value of the recurrent parent Barke for the respective trait.
