# Supplementary material for: A nested association mapping population identifies multiple small effect QTL conferring resistance against net blotch (Pyrenophora teres f. teres) in wild barley
Source: PLoS One. 2017 Oct 26;12(10):e0186803. doi: 10.1371/journal.pone.0186803 (PMC5658061; doi:10.1371/journal.pone.0186803)
Supplement: S5 File — (PDF) [file pone.0186803.s005.PDF]

Supplementary File 5

A

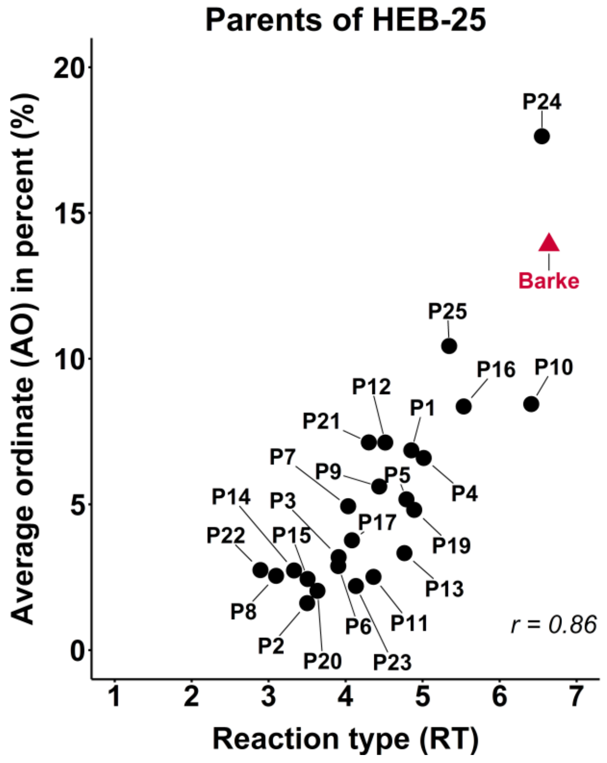

B

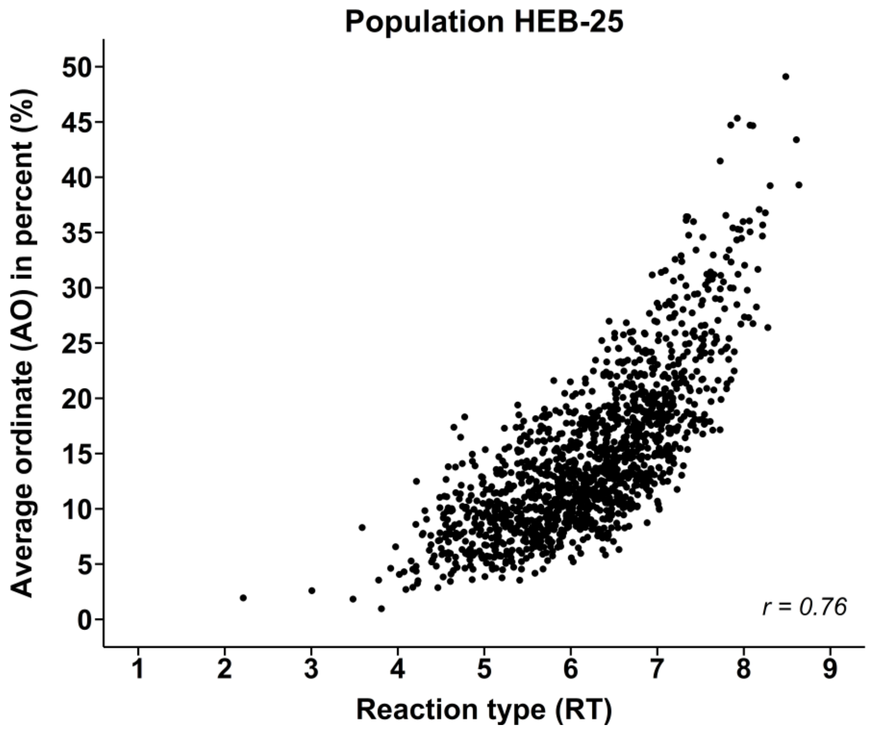

Correlation between trait average ordinate (AO) and reaction type (RT) based on two-year lsmeans of HEB-25 parents **(A)** and all HEB-25 lines **(B)**.
